# The Pro-Inflammatory Cytokine IL-22 Up-Regulates Keratin 17 Expression in Keratinocytes via STAT3 and ERK1/2

**DOI:** 10.1371/journal.pone.0040797

**Published:** 2012-07-13

**Authors:** Wei Zhang, Erle Dang, Xiaowei Shi, Liang Jin, Zhenzhen Feng, Lei Hu, Yan Wu, Gang Wang

**Affiliations:** Department of Dermatology, Fourth Military Medical University, Xijing Hospital, Xi’an, People’s Republic of China; French National Centre for Scientific Research, France

## Abstract

**Background:**

To investigate the regulation of K17 expression by the pro-inflammatory cytokine IL-22 in keratinocytes and its important role in our previously hypothesized “K17/T cell/cytokine autoimmune loop” in psoriasis.

**Materials and Methods:**

K17 expression was examined in the IL-22-treated keratinocytes by real-time quantitative PCR, ELISA, Western blot and Immunofluorescence. In addition, the signaling pathways involved in K17 regulation were investigated with related inhibitors and siRNAs. In addition, K17 expression was examined in the epidermis of IL-22-injected mouse skin.

**Results:**

IL-22-induced K17 expression was confirmed in keratinocytes and the epidermis of IL-22-injected mouse skin at both mRNA and protein levels, which is an important complement to the autoimmune loop. We further investigated the regulatory mechanisms and found that both STAT3 and ERK1/2 were involved in the up-regulation of K17 expression induced by IL-22.

**Conclusion:**

IL-22 up-regulates K17 expression in keratinocytes in a dose-dependent manner through STAT3- and ERK1/2-dependent mechanisms. These findings indicated that IL-22 was also involved in the K17/T cell/cytokine autoimmune loop and may play an important role in the progression of psoriasis.

## Introduction

Psoriasis is a chronic inflammatory skin disease that affects approximately 2% of the general population [1], [2]. It is generally believed that psoriasis is a T cell-mediated autoimmune disease in which the IL-23/Th17 pathway and the Th17-associated cytokines, IL-17 and IL-22, are considered to be involved [3], [4], [5]. IL-22, a member of the IL-10 cytokine family, signals through the class ΙΙ cytokine receptor heterodimer IL-22R/IL-10R2, which is expressed in a variety of epithelial tissues [6]. IL-22 is preferentially produced by Th17 cells and promotes keratinocyte proliferation while inhibiting differentiation [7], [8], [9]. Elevated IL-22 expression is found in the serum and skin lesions of psoriasis patients and is correlated with the severity of the disease [4]. This evidence strongly suggests that IL-22 plays a critical role in the pathogenesis of psoriasis.

Previous work indicated that a K17/T cell/cytokine autoimmune loop may exist to drive the pathogenesis of psoriasis [10], [11]. K17 is a myoepithelial keratin and is overexpressed in wound healing and in psoriatic skin lesions as compared to normal human skin [9]. Moreover, K17 expression correlates with psoriasis severity and is considered to be a hallmark of psoriasis [12]. Our previous studies demonstrated that K17 contained some restricted T cell epitopes which may promote the proliferation of psoriatic T cells and induce IFN-γ and IL-17 production [13]. IFN-γ and IL-17 up-regulate K17 expression by activating STAT1 [14] and STAT1/3[15], respectively. Thus, Th17 cells are a critical component of the K17/T cell/cytokine autoimmune loop. In addition, IL-22, preferentially produced by Th17 cells, has a strong ability to induce keratinocyte proliferation. Therefore, we hypothesized that IL-22 may be a key cytokine of the K17/T cell/cytokine autoimmune loop and induce K17 expression by activating specific signaling pathways, and thereby participate in the development of psoriasis. In the present study, we verified this hypothesis by observing the effect of IL-22 on the expression of K17 in HaCaT human keratinocytes and the epidermis of mouse skin.

## Results

### IL-22 up-regulated K17 Expression in Keratinocytes in a Dose-dependent Manner

**Figure 1:**
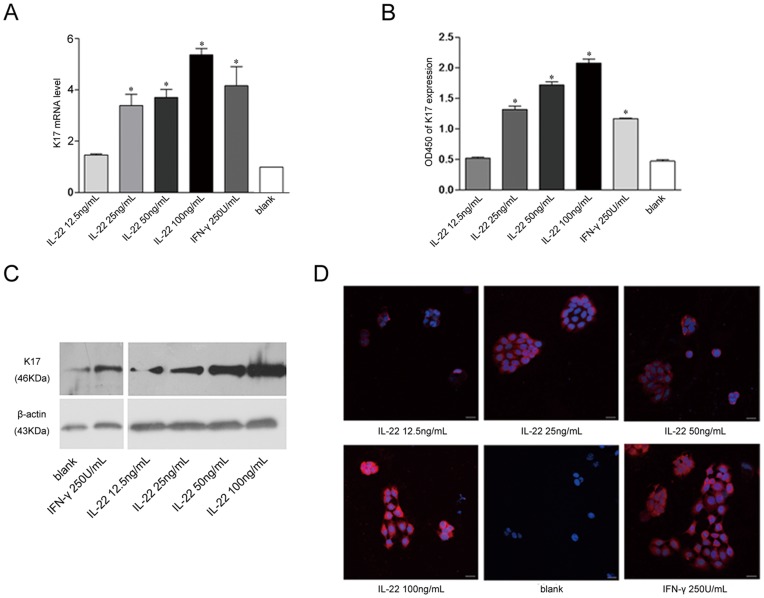
The up-regulation of K17 expression in IL-22-induced keratinocytes. (**A**) The real-time PCR analysis of K17 mRNA levels. Data are expressed as 2^−ΔΔCT^ relative to untreated HaCaT cells. (**B**) The ELISA analysis of K17 expression. (**C**) The Western blot analysis of K17 protein expression. (**D**) Immunofluorescence was performed on HaCaT cells to measure K17 expression. DAPI staining for nuclei is in blue. The scale bars represent 30 µm. The blank group is untreated HaCaT cells. Results represent means±SEM from three independent experiments. *P<0.05 was considered significant for the IL-22 treated group versus blank.

To clarify the relationship between IL-22 and K17 expression, real-time PCR was employed to detect the K17 mRNA level after (12.5, 25, 50 and 100 ng/ml) IL-22 stimulation. We found that K17 mRNA levels increased with IL-22 concentration in a dose-dependent manner, especially at higher concentration (100 ng/ml), as compared with the level in untreated cells (Fig. 1A). No significant increase in K17 mRNA expression was detected in response to the 12.5 ng/ml IL-22 treatment (P>0.05). To further confirm this finding, ELISA and Western blot assays were employed to measure K17 protein expression after IL-22 treatment of HaCaT cells for 48 h (Fig. 1B, C). K17 protein expression was up-regulated by IL-22 at concentrations of 25 ng/ml or higher. However, no significant difference in the expression levels of K17 protein was observed when the concentration of IL-22 was lower than12.5 ng/ml. Two-color immunofluorescence staining of K17 revealed weak K17 staining in the cytoplasm of untreated cells. The intracellular K17 staining intensity increased with the concentration of IL-22 stimulation after 48 hours. In particular, the K17 expression in HaCaT cells treated with 100 ng/ml IL-22 was much higher than in HaCaT cells treated with IFN-γ (Fig. 1D). Taken together, IL-22 up-regulates K17 expression in keratinocytes in a dose-dependent manner.

### STAT3 and ERK1/2 Signaling Pathways were Activated in IL-22-treated HaCaT Cells

**Figure 2:**
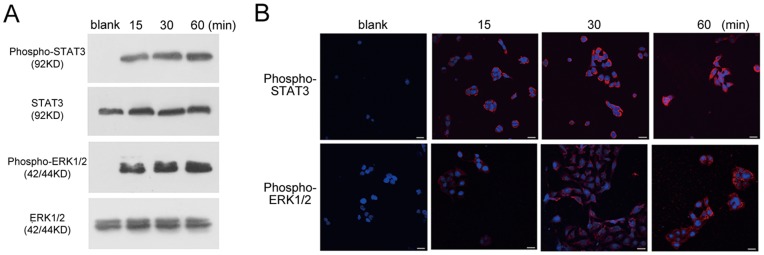
The activation of STAT3 and ERK1/2 signaling pathways in IL-22-treated HaCaT cells. HaCaT cells were treated with IL-22 and the expression of STAT3, ERK1/2, phospho-STAT3 or phospho-ERK1/2 was tested with corresponding antibodies. (**A**) The Western blot analysis of the activation of phospho-STAT3 and phospho-ERK1/2 in IL-22-treated HaCaT cells. (**B**) Immunofluorescence staining of phospho-STAT3 and phospho-ERK1/2 in IL-22-treated-HaCaT cells at different time points. Note that stronger signals were observed in the cultures at 15 min, 30 min or 60 min following IL-22 treatment. DAPI staining for nuclei is in blue. The scale bars represent 30 µm. The blank group is untreated HaCaT cells.

Because K17 expression in HaCaT cells is primarily regulated by STAT-dependent signaling pathways [15], [16], [17], the phosphorylation of STAT3 and ERK1/2 was examined in IL-22-treated HaCaT cells. Western blot analysis showed that IL-22 promoted the tyrosine phosphorylation of STAT3 and ERK1/2 from 15 to 60 minutes after IL-22 (25 ng/ml) stimulation (Fig. 2A). Immunofluorescence staining revealed similar findings. The fluorescence of activated STAT3 and ERK1/2 under confocal fluorescence microscopy gradually increased from 15 to 60 minutes after IL-22 (25 ng/ml) stimulation (Fig. 2B). These results indicated that IL-22 can activate the STAT3 and ERK1/2 pathways in HaCaT cells.

### STAT3 and ERK1/2 Antagonists and siRNA Partially Suppressed K17 Expression in HaCaT Cells

**Figure 3:**
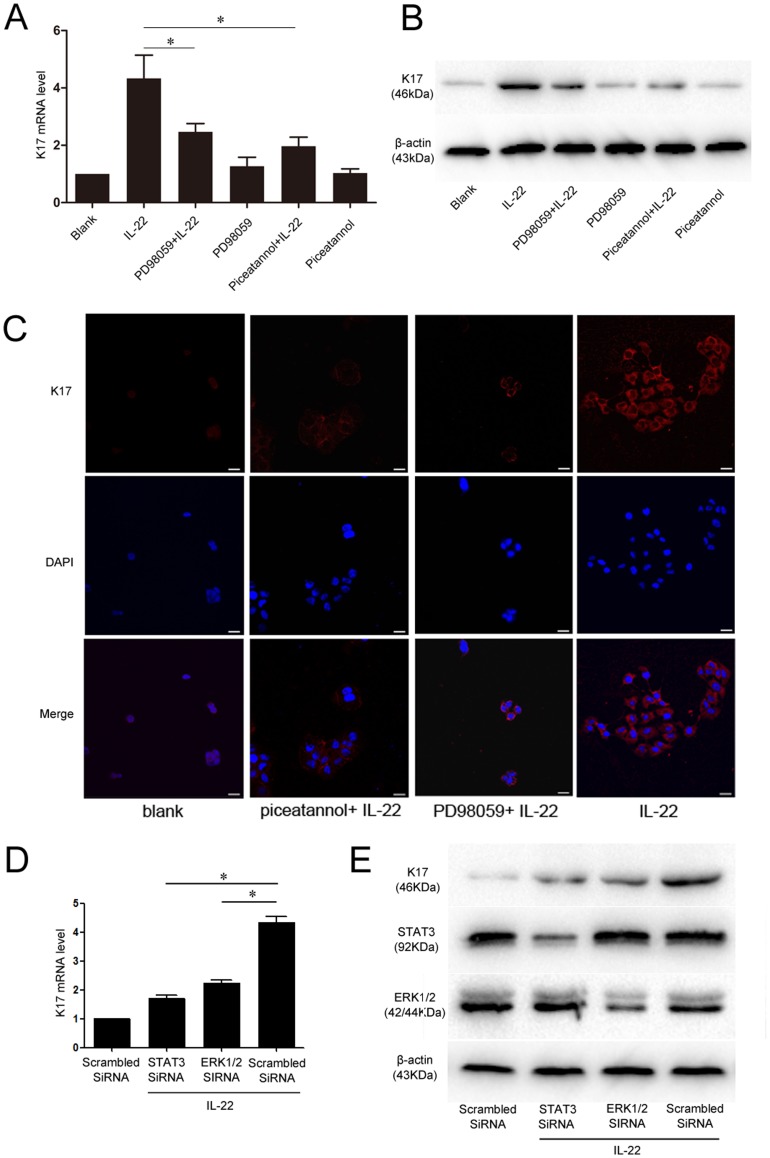
Inhibition of STAT3 and ERK1/2 signaling pathways partially suppresses the effect of IL-22 on K17 expression. (**A**) An examination of the inhibitory effect of piceatannol and on PD98059K17 mRNA levels with real-time PCR analysis. (**B**) The Western blot analysis of the inhibitory effect of piceatannol and PD98059 on K17 protein expression. (**C**) Immunofluorescence staining of K17 expression in piceatannol and PD98059-treated, IL-22 stimulated HaCaT cells, or untreated HaCaT cells. DAPI staining for nuclei is in blue. The scale bars represent 30 µm. (**D**) An examination of the inhibitory effect of STAT3 and ERK1/2 siRNA on K17 mRNA levels with real-time PCR analysis. (**E**) The Western blot analysis of the inhibitory effect of STAT3 and ERK1/2 siRNA on K17 protein expression. The blank group is untreated HaCaT cells. *P<0.05 was considered significant.

To characterize the signaling pathways by which IL-22 increases K17 expression in HaCaT cells, piceatannol and PD-98059 were used to selectively inhibit STAT3 and ERK1/2 signaling, respectively. The pre-incubation of HaCaT cells with piceatannol and PD-98059 partially suppressed the effect of IL-22 on K17 expression. A significant decrease in IL-22-induced K17 mRNA levels was found using RT-PCR analysis with STAT3 or ERK1/2 inhibition when compared with untreated groups (Fig. 3A). Western blot analysis confirmed the effects of partial inhibition on IL-22-induced K17 up-regulation in antagonist-pretreated keratinocytes (Fig. 3B). Immunofluorescence staining for K17 revealed significantly less signals in antagonist-pretreated cells after IL-22 stimulation, indicating a reduced K17 production in comparison to the untreated group (Fig. 3C). STAT3 and ERK1/2 knocked down by small interfering RNA (siRNA) before IL-22 stimulation prevented K17 induction similarly to the specific antagonists (Fig. 3D, E). In summary, STAT3 and ERK1/2 specific antagonists (piceatannol and PD-98059) and siRNA partially suppressed the effect of IL-22 on K17 expression at the mRNA and protein levels.

### Epidermal Proliferation and K17 Expression were Induced by IL-22 in vivo

**Figure 4:**
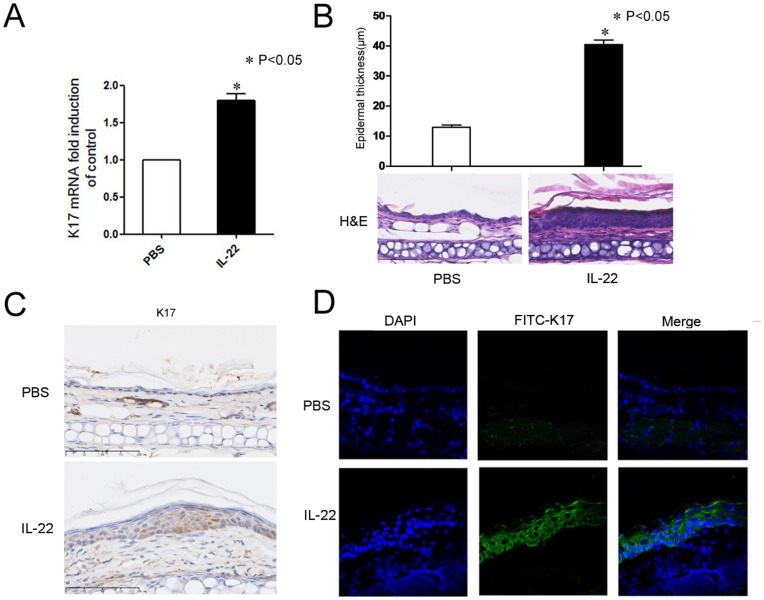
The epidermal proliferation and up-regulation of K17 induced by IL-22 in the mouse skin. (**A**) The real-time PCR analysis of K17 mRNA levels. The results represent means±SEM from three independent experiments. (**B**) H&E-stained sections of PBS- or IL-22-injected ears after daily injection for seven days. The epidermal thickness was measured. Data are from 2 experiments with 5 mice per group. (**C**) Histological and immunohistochemical analysis of K17 stimulated or not with 20 ng/ml IL-22. 4 µm vertical sections were reacted with rabbit anti-K17 then photographed under a microscope (magnification×400). (**D**) Frozen sections of ears from PBS-injected or IL-22-injected mice stained with rabbit anti-mouse K17 followed by FITC-conjugated goat anti-rabbit IgG (green). DAPI staining for nuclei is blue. *P<0.05 was considered significant for the IL-22 injected mouse group versus PBS control.

An *in vivo* experiment was performed to confirm that IL-22 can up-regulate K17 expression in keratinocytes. We examined whether IL-22 increased the expression of K17 in keratinocytes after IL-22 injection in mouse skin using RT-PCR and immunohistological analyses. The expression of K17 mRNA after two IL-22 injections was 1.79-fold higher than that of the control group (Fig. 4A). H&E staining of sections from mouse ears after seven daily injections showed epidermal acanthosis (thickening of the spinous layer). The epidermal thickness was 40.48±1.53 µm in the IL-22 group and 12.94±0.76 µm in the control group (P<0.05). Additionally, fluorescent microscopic analysis with anti-K17 revealed the prominent up-regulation of K17 in the IL-22-treated, acanthotic mouse epidermis (Fig. 4B, C, D). These results suggest that IL-22 can also up-regulate K17 expression and result in acanthosis *in vivo*.

### IL-17A and IL-22 or IFN-γ Synergistically Induced K17 Expression in Keratinocytes

**Figure 5:**
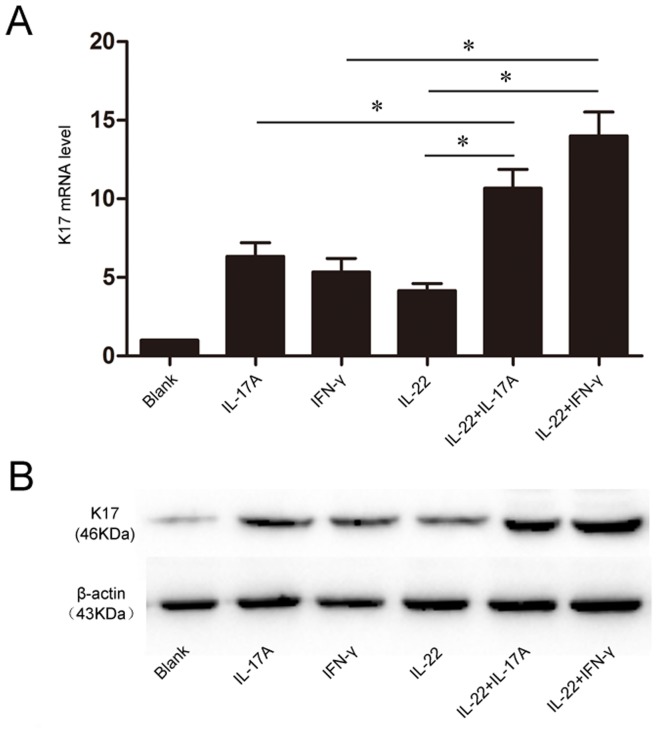
The synergism of IL-22, IL-17A and IFN-γ in inducing K17 expression. HaCaT were treated with IL-22(25 ng/ml), IL-17A (100 U/ml) and IFN-γ (100 U/ml) alone or in combination. (**A**) The real-time PCR analysis of K17 mRNA levels after 24 h; Data are expressed as 2^−ΔΔCT^ relative to untreated HaCaT cells. (**B**) The Western blot analysis of K17 protein expression. The blank group is untreated HaCaT cells. Results represent means±SEM from three independent experiments. *P<0.05 was considered significant.

So far, both IL-17A and IL-22 reported by our group could induce the K17 expression besides IFN-γ. However, the interactions of these cytokines in the process were still ambiguous. Thus, we observed the effect of Th17 cytokine IL-22, IL-17A and IFN-γ, alone or in combination on inducing K17 expression in HaCaT. Compared with the single stimulation, IL-22 and IL-17A or IFN-γ can synergistically up-regulate K17 expression at both mRNA and protein level (Fig. 5A, B). Those results suggested IL-22 could synergetically help IL-17A and IFN-γ to induce K17 expression in HaCaT.

## Discussion

In this study, we investigated the relationship between IL-22 and K17 and demonstrated that K17 expression could be induced by IL-22 in keratinocytes in a dose-dependent manner via STAT3 and ERK1/2 signaling pathways both *in vitro* and *in vivo*. The IL-22 signaling pathway involves the JAK/STAT and MAPK networks, among others [18], [19], [20]. Our findings indicate that HaCaT cells treated with IL-22 exhibited an increased phosphorylation of STAT3 and ERK1/2 as compared to control cells. In experiments using specific antagonist of STAT3 and ERK1/2 or siRNA, the IL-22 induction of K17 mRNA and protein expression significantly decreased, indicating that the antagonists and siRNA successfully blocked the cell signaling involved in K17 expression, *i.e.*, that IL-22 induced K17 expression in HaCaT cells through STAT3 and ERK1/2. STAT3 and ERK1/2 are known to be closely associated with psoriasis. A molecule known to be involved in Th17 signaling, STAT3 was found to be highly expressed in keratinocytes in psoriatic skin lesions, especially in the nucleus, where it participates in the hyper-proliferation of keratinocytes and inflammatory infiltration [21]. In addition, extensive ERK1/2 phosphorylation was found in the nuclei of keratinocytes in the stratum basale and stratum spinosum of psoriatic skin lesions, correlating strongly with the hyper-proliferation of keratinocytes in psoriasis [22], [23], [24]. Additionally, ERK1/2 phosphorylation has an activating, pro-proliferative and anti-apoptotic effect in T cells [25]. Therefore, our finding that IL-22 induced K17 expression in HaCaT cells by activating STAT3 and ERK1/2 signaling pathways is consistent with the role that these two pathways play in the pathogenesis of psoriasis.

Th17 cells are important regulators in the pathogenesis of psoriasis [26]. As their effector molecule, IL-22 positively correlates with the severity of psoriasis and is highly expressed in the serum and skin lesions of psoriasis patients [5], [27]. Therefore, the induction by IL-22 of K17 expression suggests that a “Th17/IL-22/K17 autoimmune loop” exists in psoriasis: K17 induces the activation and proliferation of T cells in psoriasis; T cells then produce IL-22, which can induce the expression of K17, forming an interacting cycle. This new loop is an important complement to the former “K17/T cell/cytokine autoimmune loop” and represents a better understanding of the relationship among cytokines, T cells and keratins in the pathogenesis of psoriasis. Other studies indicate that IL-22 regulates the expression of antimicrobial proteins, differentiation-associated proteins and mobility-regulating proteins in keratinocytes, while suppressing the differentiation of keratinocytes and resulting in psoriatic skin lesions in the epidermis [5], [28], [29]. Thus, IL-22 is considered to be an important inducer of keratinocyte proliferation in the pathogenesis of psoriasis. Our research further confirmed that IL-22, via its induction of K17 expression, is an important regulator of keratinocytes. This relationship may also be indicating that K17 is the autoantigen in psoriasis.

It has now been demonstrated that IFN-γ, IL-17, IL-22 and certain other cytokines all participate in the “T cell/cytokine/K17 autoimmune loop” by inducing K17 production. These are all psoriasis-related cytokines, and they also have close interactions with one another. IFN-γ can up-regulate IL-22R1 expression in HaCaT cells both dose-dependently and time-dependently, indicating that IFN-γ may be able to enhance the sensitivity of keratinocytes to IL-22 under inflammatory circumstances [28], [30]. IL-22 not only acts synergistically with IL-17A or IL-17F in inducing the expression of hBD2, hBD3, S100A7, S100A8 and S100A9, but also exhibits similar effects to IL-17 on keratinocytes, inducing the expression of antimicrobial peptides and CXCL8 [29], [31], [32], [33]. Our research further confirmed these similarities in findings that IL-22 and IL-17 alone or in combination can induce the proliferation of keratinocytes and the expression of K17 in the epidermis of mice, which suggests that IL-17 and IL-22 share some signaling pathways and functions in the pathogenesis of psoriasis. In addition, the Th17 cytokines IL-17 and IL-22 have synergetical effect as psoriasis-related cytokines, and the synergism of inflammatory cytokines may be an important process of the pathogenesis of psoriasis.

To better understand the pathogenesis of psoriasis, the interactions between cytokines and cells, the signaling pathways and the changes in the biological behaviors of target cells need to be investigated further. Our study proved that IL-22 can induce K17 expression in HaCaT cells in a dose-dependent manner via STAT3 and ERK1/2 signaling pathways. These findings complemented our earlier hypothesis of a “T cell/cytokine/K17 autoimmune loop” and further clarified the role of Th17 cytokines in the development of psoriasis. The pathogenesis of psoriasis is complicated at least partly because the cells and cytokines involved form an intricate, interacting network. Our research focused on the IL-22 regulation on K17 expression in HaCaT cells and in an animal model. The finding clarified the molecular mechanisms involved and helped to further understand the pathogenesis of psoriasis and lay the foundation for specific immunotherapy.

## Materials and Methods

### Ethics Statement

The animal experimental procedures were approved by the Institutional Animal Care and Use Committee of the Fourth Military Medical University (approval number: 12003). The number of animals used and their suffering were minimized.

### Cell Culture and IL-22 Treatment in vitro

The human HaCaT Keratinocyte cell line [15] was grown in Dulbecco’s Modified Eagle’s Minimal Essential Medium (DMEM) (containing 1.05 mM Calcium Chloride)(Gibco-Invitrogen, Carlsbad, CA) supplemented with 10% fetal bovine serum (Gibco-Invitrogen, Carlsbad, CA) in a humidified atmosphere containing 5% CO_2_ at 37°C. After 24 hours of FBS starvation in DMEM, cells at 60% confluence were stimulated with IL-22 (12.5, 25, 50, or 100 ng/ml) (PeprotechInc., Rocky Hill, NJ) for 48 hours, and IFN-γ (PeprotechInc., Rocky Hill, NJ) treated HaCaT cells (250 U/ml) were used as a positive control. To inhibit the STAT3 and ERK1/2 signaling pathways, piceatannol and PD-98059(100 µM each) (Sigma, Saint Louis, MO) were added 2 hours before stimulation with IL-22 (PeprotechInc., Rocky Hill, NJ).

HaCaT cells were transfected with siRNAs using Lipofectamine 2000 (Invitrogen, Carlsbad, CA), according to the manufacturer’s instructions, 24 hours before stimulation with IL-22.

### Reagents

Rabbit anti-human K17 monoclonal antibody and β-actin antibody were obtained from Abcam (Cambridge CB4 0FL, UK). Horseradish peroxidaseconjugated goat anti-rabbit antibody IgG was obtained from Zhongshan Godenbrige (Beijing, China). 4′, 6-diamidino-2-phenylindole (DAPI) and CY3-labeled goat anti-rabbit antibody IgG were obtained from KPL (Gaithersburg, Maryland, USA). Piceatannol was obtained from Sigma (Saint Louis, MO, USA). Rabbit anti-human STAT3, ERK1/2, phospho-STAT3, phospho-ERK1/2 and ERK1/2 siRNA and scrambled control siRNA were obtained from Cell Signaling Technology (Danvers, MA,USA). STAT3 siRNA were synthesized by GenePharma (Shanghai, China).

### Quantitative RT-PCR

Total RNA was extracted using TRIzol (Takara, Ohtsu, Japan) and subsequent chloroform/isopropanol/ethanol purification. A PrimeScript™ RT reagent Kit (Takara, Ohtsu, Japan) was used to convert RNA into cDNA and one micrograms of total RNA was used per well. Quantitative RT-PCR was conducted using the SYBR Premix Ex Taq™^ II^ (Takara, Ohtsu, Japan) on a Chromo4 continuous fluorescence detector with a PTC-200 DNA Engine Cycler (Bio-Rad, Hercules, CA) The K17 specific primer were: Homo sapiens keratin17 primer (forward: 5′-ACCATGCAGGCCTTGGAGA-3′ and reverse: 5′-GTCTTCACATCCAGCAGGA-3′); Mus musculus keratin17 primer (forward: 5′-ACCATGCAGGCCCTGGAGA-3′ and reverse: 5′-GTCTTCACATCCAG CAGGA-3′); β-actin (forward, 5′-CACGATGGAGGGGCCGGACTCATC-3′ and reverse, 5′-TAAAGACCTCTATGC CAACACAGT-3′). β-actin, an internal control, was used for amplification of Homo sapiens and Mus musculus. The cycling conditions were as follows: 95°C for 2 minutes followed by 45 cycles of denaturation at 95°C for 5 seconds, annealing at 60°C for 10 seconds, and extension at 72°C for 15 seconds. All reactions were run in triplicates for at least three independent experiments. Samples were normalized to the independent control housekeeping gene β-actin and were reported according to the ΔΔCT method as RNA fold increase: 2^ΔΔCT^ = 2^ΔCT sample^- 2 ^ΔCT reference^. Results were considered significant when at least a 2-fold difference in expression levels was detected.

### Western Blot Analysis

Cells were homogenized in cell lysis reagent (Runde Biologicals Ltd, Xi’an, China) and phenylmethanesulfonylfluoride (protease inhibitors mix) (Sigma-Aldrich, USA). Following centrifugation at 10,000 r/min for 10 minutes at 4°C, equal amounts of proteins were separated by a 10% SDS-PAGE and were transferred to polyvinylidene difluoride (PVDF) membranes (Invitrogen, Carlsbad, CA, USA). After blocking for 2 hours with blocking buffer (1×Tris-buffered saline with 5% skim milk and 0.01% Tween- 20), the membranes were blotted with the primary antibodies, including K17, β-actin, STAT3, ERK1/2, phospho-STAT3, phospho-ERK1/2 (Cell Signaling Technology, Danvers, MA) overnight at 4°C. Membranes were washed with TBS and then incubated with HRP-conjugated secondary antibodies (Dako Cytomation, Glostrup, Denmark) for 1h at room temperature. Proteins were detected using a chemiluminescence detection kit (KPL, Gaithersburg, MD).

### Enzyme-linked Immunosorbent Assay (ELISA)

HaCaT Cells were seeded directly into 96 well plates at a density of 1.5×10^4^/well and were stimulated with IL-22 and IFN-γ, as above, at 37°C for 24 hours. After blocking for 1 hour with blocking buffer (0.01 mol/L PBS, 10% FBS) at 37°C, the cells were treated with 0.3% Triton X-100 for 10 minutes at room temperature. After wash three times with 0.01 mol/L PBS, the cells were incubated with rabbit anti-human K17 monoclonal antibody for 1 hour at 37°C followed by incubation with the secondary horseradish peroxidase conjugated goat anti-rabbit IgG for 1 hour at room temperature. The plates were washed and developed with TMB Plus (3, 30, 50-tetramethylbenzidine plus hydrogen peroxide; Kem-En-Tec, Taastrup, Denmark). Absorbance (A450) was measured with a plate reader (MRP-2100, Syntron, USA).

### Immunofluorescence Confocal Microscopy

HaCaT Cells were grown on the coverslips in 6-well plates and then cultured with IL-22 and IFN-γ as above. The coverslips were rinsed twice with 0.01 mol/L PBS and fixed with acetone at 4°C for 10 minutes. The cells were next incubated in 0.3% Triton X-100 for 5 min at room temperature. Non-specific interactions were blocked with 4% BSA at 37°C for 30 minutes. After rinsing three times with 0.01 mol/L PBS, cells were incubated with rabbit anti-human K17 mAb, rabbit anti-human phospho-STAT3 or rabbit anti-human phospho-ERK1/2 mAb overnight at 4°C, and then with secondary CY3-labeled goat anti-rabbit IgG for 1 hour at 37°C in the dark. DAPI was applied to all cells for nuclear counterstaining. Samples were analyzed by confocal microscopy using an FV-1000/ES confocal microscope (Olympus, Tokyo, Japan).

### Animal Experiments of IL-22 Induced Epidermal Changes

Female BALB/c mice aged 8–10 weeks were obtained from the Department of Laboratory Animal Medicine of the Fourth Military Medical University. Mice were randomly assigned to experimental groups of five mice each, and each experiment was repeated at least 3 times. An intradermal injection of 20 µL PBS alone or containing 1 µg recombinant mouse IL-22 (PeprotechInc., Rocky Hill, NJ) into the ears of anesthetized mice using a 30-gauge needle was performed daily for 2 days or 7 days [34]. Twenty-four hours after the last injection, the ears were collected and frozen immediately in liquid nitrogen for RNA quantification, H&E staining and Immunofluorescence microscopy analysis. 4 µm sections of mouse ear were fixed in 10% formalin in PBS and stained with H&E. The H&E slides were digitalized with the Nanozoomer Digital Pathology System (Hamamatsu, Herrsching, Germany) at 400 × magnification. The epidermal thickness was measured based on the picture of the H&E images with NDP View Software (Hamamatsu, Herrsching, Germany). Frozen sections of mouse ear were prepared for immunoﬂuorescence staining. Sections were fixed with chilled acetone for 10 min, blocked for 30 min at room temperature with 5% goat serum in PBS, and incubated with rabbit anti-mouse K17 pAb (Abcam, Cambridge, UK) for 1 hour at room temperature. After washing in PBS, sections were incubated for 30 minutes at room temperature with FITC-conjugated goat-anti-rabbit IgG (KPL, Gaithersburg, MD), washed in PBS and counterstained with DAPI nuclear stain (KPL, Gaithersburg, MD). Laser scanning confocal microscopy was performed with a FV-1000/ES confocal microscope (Olympus, Tokyo, Japan).

### Statistical Analyses

All the experiments had been repeated three times at least and the statistical analyses were performed using GraphPad Prism 5.0(GraphPad Software, San Diego, CA). For experiments with more than two groups, the differences between groups were compared by a one-way analysis of variance (ANOVA) followed by Dunnett’s test, in which all groups were tested against a control group as a reference. For experiments with only two groups, Student’s t-test was used for comparisons of group means. P-values <0.05 were considered to represent significant differences.
